# The Medical World

**Published:** 1857-01

**Authors:** 


### EDITORIAL.

*The Medical World.*

For some time we have been receiving a weekly paper bearing this title, and edited by J. V. C. Smith, M.D. formerly editor of the *Boston Medical and Surgical Journal.* On looking over the first numbers, we were satisfied that it was little else than an advertising sheet for quacks and quackery, and, consequently, neither worthy of a place on our exchange list, nor of sufficient importance to require a notice. We have seen nothing in the later numbers to cause any change in our opinion; but, as most of our editorial brethren have deemed it their duty specially to condemn the hybrid sheet, we feared that entire silence on our part might be misconstrued.

Under the pretence of greater *liberality*, Dr. Smith has undertaken to edit a sheet, the columns of which are open alike to all the *isms* and *pathys* of the day; and which is regularly enclosed in a wrapper filled with advertisements of patented and quack nostrums. Of course it cannot receive the patronage or favor of any well-informed physician. We can account for Dr. Smith's course in attempting to edit such a periodical, only on the supposition that, while gyrating in the dirty whirlpool of politics during the last three or four years, his head has become so giddy that he is unable to discern clearly the difference between the action of a Homœpathic pellet of sugar of milk and a regular Hydropathic *packing*.

That something has exerted a sad degree of demoralizing influence on the man, is further evident from the scurrilous language and malicious sentiments which he uttered in relation to the able and fearless editor of the *Buffalo Medical and Surgical Journal*. Dr. Hunt, of the *Buffalo Journal*, needs no defence, however; for to be abused in such a sheet as the *Medical World*, is equivalent to a high compliment from the profession at large, while to be praised would justly excite the suspicions of the upright.

By the way, we notice in the *Medical World* a special disposition to puff the Medical Department of the University of Michigan. Is there any truth in the old adage, 'Birds of a feather will flock together'?

